# Spatial correction improves accuracy of catheter positioning during ablation of premature ventricular contractions: differences between ventricular outflow tracts and other localizations

**DOI:** 10.1186/s12872-022-02741-3

**Published:** 2022-07-13

**Authors:** M. Nies, R. Schleberger, L. Dinshaw, N. Klatt, P. Muenkler, C. Jungen, L. Rottner, M. D. Lemoine, B. Reißmann, A. Rillig, A. Metzner, P. Kirchhof, C. Meyer

**Affiliations:** 1 Department of Cardiology, University Heart and Vascular Center, University Medical Center Hamburg-Eppendorf, Martinistraße 52, 20246 Hamburg, Germany; 2 DZHK (German Center for Cardiovascular Research), Partner Site Hamburg/Lübeck/Kiel, Berlin, Germany; 3 Department of Cardiology, Schön Klinik Neustadt in Holstein, Am Kiebitzberg 10, 23730 Neustadt in Holstein, Germany; 4 Department of Cardiology, Leiden University Medical Center, Albinusdreef 2, 2333 ZA Leiden, The Netherlands; 5 Institute of Cardiovascular Sciences, University of Birmingham, Birmingham, UK; 6 Division of Cardiology, EVK Düsseldorf, cNEP, Cardiac Neuro- and Electrophysiology Research Consortium, Kirchfeldstraße 40, 40217 Düsseldorf, Germany; 7 cNEP, Cardiac Neuro- and Electrophysiology Research Consortium, Institute for Neural and Sensory Physiology, Heinrich Heine University Düsseldorf, Medical Faculty, Düsseldorf, Germany

**Keywords:** Hybrid mapping, Premature ventricular contractions, Activation mapping, Catheter ablation

## Abstract

**Background:**

Hybrid activation mapping is a novel tool to correct for spatial displacement of the mapping catheter due to asymmetrical contraction of myocardium during premature ventricular contractions (PVC). The aim of this study is to describe and improve our understanding of spatial displacement during PVC mapping as well as options for correction using hybrid activation mapping.

**Methods and results:**

We analyzed 5798 hybrid mapping points in 40 acquired hybrid maps of 22 consecutive patients (age 63 ± 16 years, 45% female) treated for premature ventricular contractions (PVCs). Median PVC-coupling interval was 552 ms (IQR 83 ms). Spatial displacement was determined by measuring the dislocation of the catheter tip during PVC compared to the preceding sinus beat. Mean spatial displacement was 3.8 ± 1.5 mm for all maps. The displacement was 1.3 ± 0.4 mm larger for PVCs with non-outflow-tract origin compared to PVCs originating from the ventricular outflow tracts (RVOT/LVOT; p = 0.045). Demographic parameters, PVC-coupling-interval and chamber of origin had no significant influence on the extent of spatial displacement.

**Conclusion:**

Ectopic activation of the ventricular myocardium during PVCs results in spatial displacement of mapping points that is significantly larger for PVCs with non-outflow-tract origin. The correction for spatial displacement may improve accuracy of radiofrequency current (RFC)-application in catheter ablation of PVCs.

## Introduction

During the last decades, catheter ablation of premature ventricular contractions (PVCs) has developed into a standard treatment for symptomatic patients [1–4]. Technical and procedural advances have improved outcome and safety so far that catheter ablation is now recommended as primary treatment option for outflow tract PVCs [5]. In light of these developments, the role of catheter ablation in clinical practice is expected to increase in the near future.

To guide the catheter for mapping and ablation, three-dimensional, electroanatomic mapping of PVCs is a routine method during interventional treatment [6].

During PVCs, the ectopic origin of myocardial activation results in an asymmetrical contraction sequence, commencing during the diastolic phase of the cardiac cycle. Depending on the contraction sequence and the time of PVC-onset, a spatial shift of myocardial tissue occurs during PVC compared to its location during normal sinus rhythm, as it has first been described by Andreu et al. [7]. In conventional activation mapping, the location of each mapping point is recorded after the predefined pattern in surface ECG was matched to the PVC-morphology. It therefore represents the catheter position after complete myocardial activation. Since catheter ablation is usually performed during sinus rhythm, this phenomenon can lead to imprecise localization of ablation targets. Correcting for this shift may facilitate the precise localization of the origin of PVCs and therefore allow to direct radio-frequency-current (RFC)-impulses with higher accuracy.

A novel mapping software tool (CARTO III, Software Version 7 Carto Prime, Biosense Webster) integrates an algorithm for correction of the aforementioned shift: for each registered PVC, the electrogram during the ectopic beat is paired with the location of the preceding sinus beat. This novel mapping algorithm is referred to as “hybrid mapping” [8]. Recently, first clinical results have been published for mapping and ablation with correction for the spatial displacement [8, 9]. However, more data is needed to evaluate the extent and underlying mechanisms of the myocardial shift. Patient-specific factors and anatomical characteristics of different myocardial areas might affect spatial displacement. The aim of this study is to describe the extent and influencing factors of spatial displacement in hybrid activation mapping of PVCs.

## Materials and methods

### Patient selection

For this monocentric study, all patients who underwent catheter ablation for PVCs between April 2019 and April 2020 using the novel mapping feature were analyzed. Procedures, in which pacemapping was used due to insufficient intraprocedural incidence of PVCs, were excluded. Clinical characteristics were obtained by review of the medical records and charts. Structural heart disease was defined as coronary artery disease leading to interventional treatment, history of myocarditis, significant valvular disease leading to ventricular dysfunction, dilated/hypertrophic cardiomyopathy or systemic disease with cardiac manifestation (e.g. sarcoidosis).

### Mapping and ablation

Ablation procedures were performed under conscious sedation using propofol and fentanyl. Orciprenaline was administered to provoke PVCs during ablation procedures when required. Catheters were positioned via femoral venous and/or retrograde arterial access depending on the respective chamber of interest. Systemic heparinization to achieve an activated clotting time of 250–300 s was performed for left-sided procedures.

Three-dimensional mapping was performed using CARTO III (Software Version 7 Carto Prime, Biosense Webster, Diamond Bar, CA, USA) with the integrated hybrid mapping module: Both the clinical PVC and a normal sinus beat were saved as ECG-pattern. When a PVC matching the predefined pattern was recorded, the local activation time (LAT) during the PVC was projected onto the catheter position recorded during the preceding sinus beat. In that way, catheter movement due to unphysiological contraction is corrected and the mapping point represents the position of the corresponding myocardium in sinus rhythm. The distance between catheter location during PVC and the location during the preceding sinus beat was defined as spatial displacement. The threshold for matching a PVC to the predefined pattern was set to 98%. As reference for local activation time, a precordial lead with a well-defined, stable R-peak during PVCs was selected. Standard catheter for mapping and ablation was a 3.5 mm irrigated tip catheter (Carto NaviStar ThermoCool, 8 French, D-Curve, Biosense Webster). Ablation was performed in the area of earliest activation using radiofrequency current with a power between 20 and 50 W depending on the target area. Adequate lesion formation was secured by monitoring of local impedance. Contact force was not used routinely as it can have a slight but sometimes significant increase in catheter stiffness in our experience.

### Quantification of spatial displacement

During the ablation procedure, we carefully reviewed the annotations to obtain correct data for spatial displacement. Offline, all non-hybrid points and floating points were deleted in order to create a map comprised exclusively of hybrid points. For each point, spatial displacement and PVC-coupling interval were analyzed. Additionally, the mean spatial displacement was calculated for all recorded hybrid points. In each map, the exact location of PVC-origin, chamber of PVC-origin, number of mapping points, number of hybrid mapping points and median spatial displacement were analyzed. The timing of PVC-onset during the cardiac cycle was represented by median PVC-coupling interval as this period is most relevant for the acquisition of hybrid mapping points.

### Statistical analysis

Descriptive statistics are presented as count and percentage for categorical and ordinal variables and as mean ± standard deviation for continuous variables if normally distributed, and as median (interquartile range) otherwise.

To analyze the association between spatial displacement and potential influencing variables, linear regressions were calculated using a mixed effects model to account for repeat measurements (multiple maps) in several cases. For regression analyses, maps of the great cardiac vein and aorta were excluded. Demographic parameters, antiarrhythmic medication, mapped cardiac chamber, left-ventricular ejection fraction, origin of PVCs and median coupling interval were defined as fixed effects. Patient-ID was defined as random effect. The regression coefficient calculated in the linear mixed effects model was used to determine the alteration of spatial displacement depending on the change of the predictive variable. Two-sided p < 0.05 were considered statistically significant. The reported p-values are used as descriptive measures only. All statistical calculations were performed in IBM SPSS Version 26.0.0.0.

## Results

**Fig. 1:**
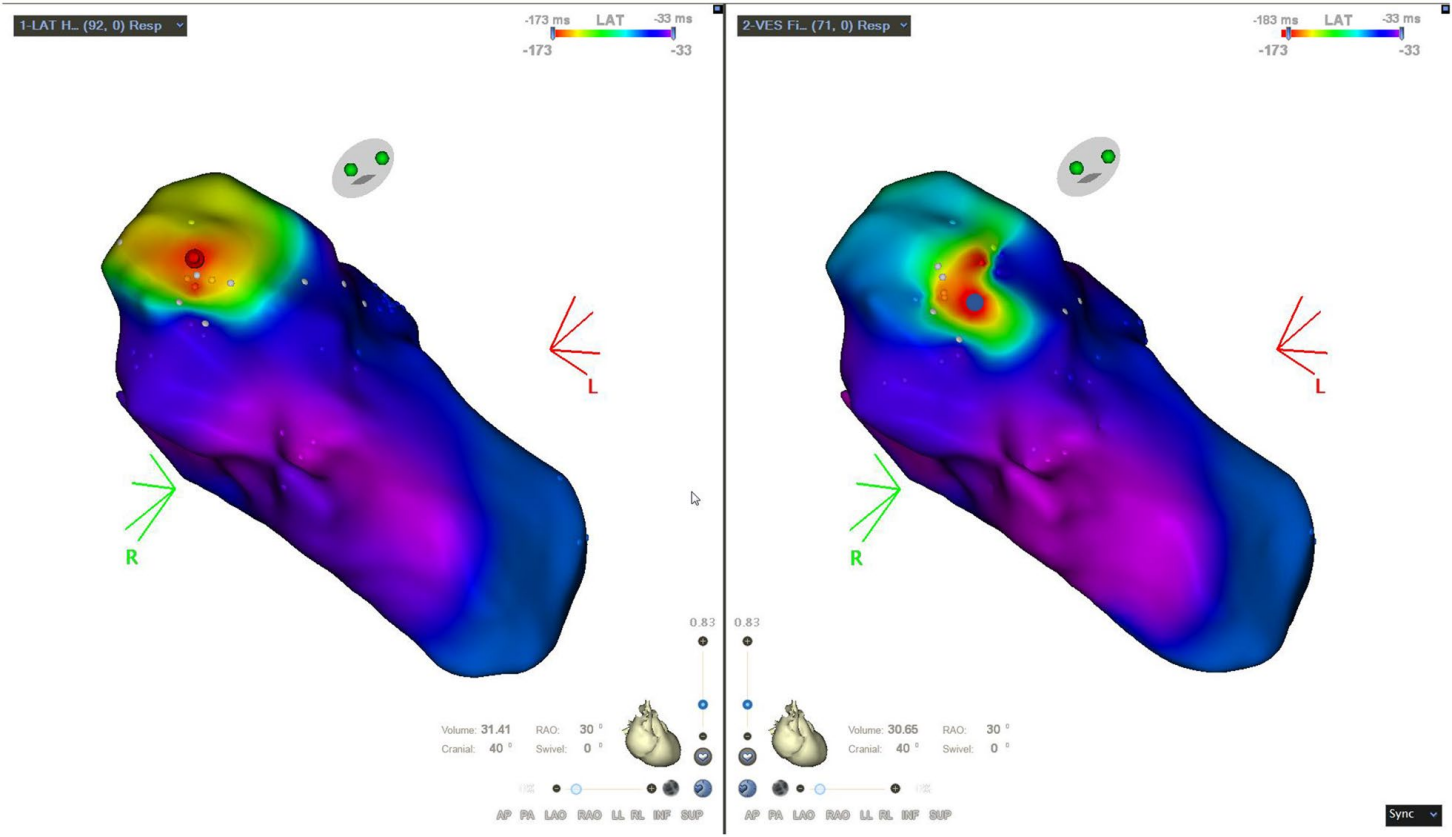
Visualization of spatial displacement. Activation maps of PVCs using CARTO III, Software Version 7 Carto Prime, Biosense Webster, RAO-view (45°). Left panel: Conventional activation map during an ablation procedure of PVCs with origin in the outflow tract. Right panel: Activation map with correction for spatial displacement for the same PVC-morphology. Red point: Mapping point with earliest local activation time (LAT) in the conventional map. Blue point: Mapping point with earliest LAT in hybrid activation map. Colors indicate the LAT relative to the selected reference in surface ECG. Red areas show the earliest activation, while late activation is visualized as purple area (see LAT scale in the top right corner). In this case, the area of earliest local activation was spatially displaced by 7.5 mm compared to the conventional activation map

Baseline parameters are shown in Table 1. Twenty-two patients were included in the study (55% male, age 63 ± 16 years). Twenty-four ablation procedures were performed using hybrid mapping. In two cases, a second ablation procedure was performed: One patient with cardiac sarcoidosis developed PVCs of a different morphology that was treated via catheter ablation 9 months after the first procedure. Another patient showed PVCs originating close to the His-bundle. After careful RFC-application and initial suppression of PVC in the first procedure, early recurrence of the targeted PVCs necessitated a second catheter ablation, which resulted in lasting suppression of PVCs. In all other cases, acute suppression of PVCs was achieved. In total, 40 three-dimensional maps containing 5798 hybrid points were analyzed. An example for the impact of the novel mapping modality is shown in Fig. 1. Twenty-four maps (60%) were recorded in the left ventricle (LV), 12 maps (30%) in the right ventricle (RV), 2 maps (5%) in the great cardiac vein and 2 maps (5%) in the proximal aorta. In 12 procedures (50%), the origin of the mapped PVC-morphology was confirmed in the ventricular outflow-tracts (6 RVOT, 6 LVOT, outflow-tract PVCs). Non-outflow tract PVCs originated from the LV in 9 cases (39%; high LV-septum: 3, inferoseptal LV: 2, free LV-wall: 1, posteromedial papillary muscle: 1, posterior mitral annulus: 1, septal LV: 1) and from the basal RV in 1 case. Two patients (8%) showed PVCs with an epicardial origin, which were treated by ablation via the great cardiac vein. Mean correction for spatial displacement was 3.8 ± 1.5 mm for all mapping points. Median PVC-coupling interval was 552 ms (IQR 83 ms).

**Table 1:** Baseline parameters

Characteristics	Total (n = 22)
Sex	
Male	12 (55%)
Age	63 ± 16 years
Structural heart disease	13 (59%)
Coronary artery disease	9 (41%)
Dilated cardiomyopathy	2 (9%)
Cardiac sarcoidosis	1 (5%)
History of myocarditis	1 (5%)
Impaired ejection fraction	12 (55%)
Mild	6 (27%)
Moderate	4 (18%)
Severe	2 (9%)
PVC-burden in 24 h-Holter	26.2 ± 15.8%
Clinical symptoms	
Dyspnea	14 (64%)
Palpitations	14 (64%)
Syncope	1 (5%)
Cardiovascular risk factors	
Hypertension	12 (55%)
Diabetes mellitus	4 (18%)
Chronic kidney disease	7 (32%)
Prior myocardial infarction	5 (23%)
BMI	27.4 ± 4.5
Prior ventricular ablation	3 (15%)
Antiarrhythmic medication	
Betablocker	1 (5%)
Flecainide	17 (77%)

Baseline parameters: Categorial and ordinal parameters are displayed as absolute number, relative proportion in parentheses. Continuous parameters are shown as mean ± standard deviation

**Fig. 2:**
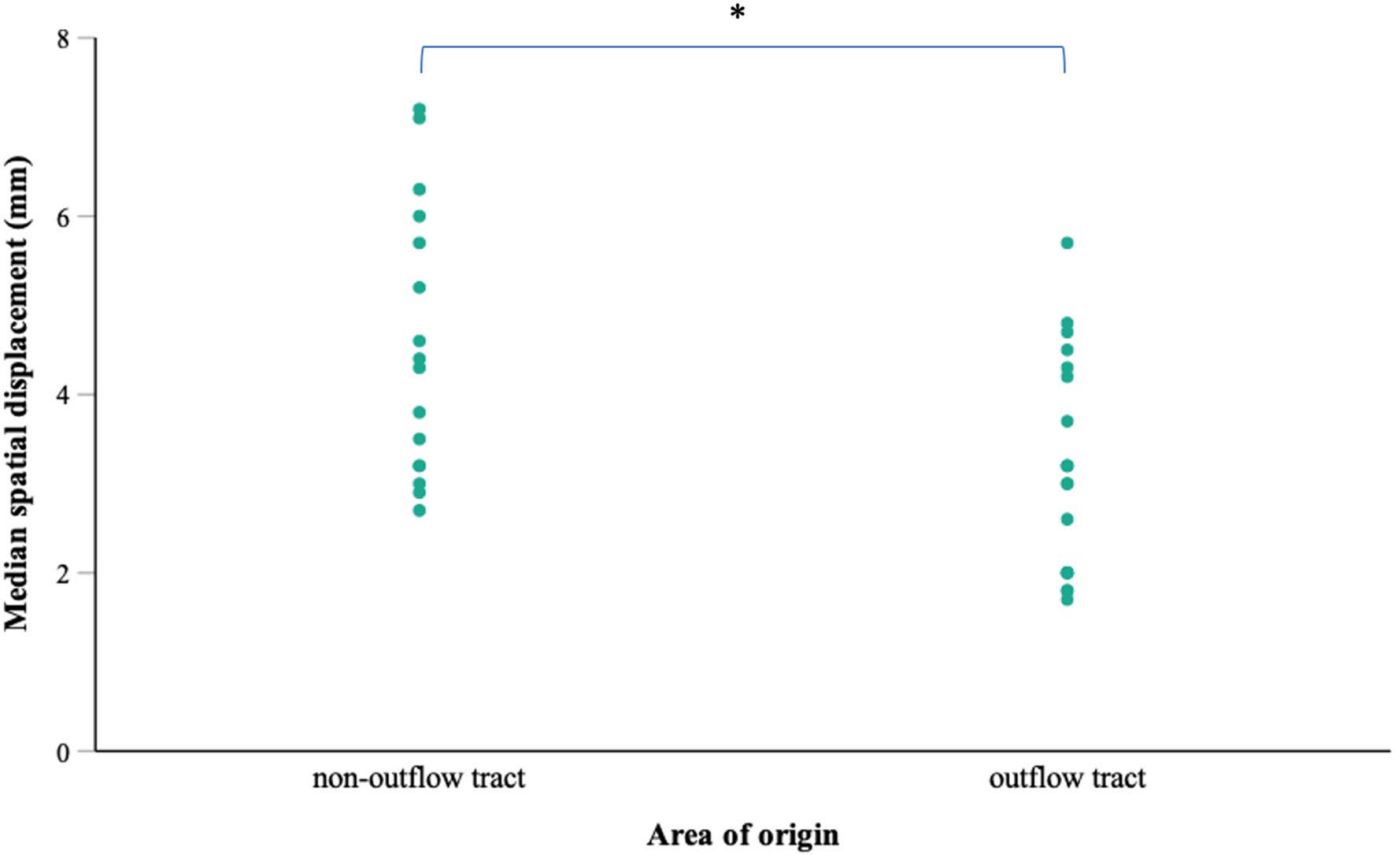
Influence of PVC-origin on spatial displacement. Scatter plot showing spatial displacement of mapping points depending on the area of PVC-origin. The median spatial displacement was significantly lower in maps recorded for PVCs originating in the ventricular outflow tracts (p = 0.045). Standard deviation was comparable in both groups

On average, the spatial displacement of mapping points was 1.3 ± 0.4 mm larger for non-outflow-tract PVCs (4.5 ± 1.5 mm vs. 3.2 ± 1.2 mm; p = 0.045 in linear mixed effects model). The impact of the site of PVC-origin on spatial displacement is shown in Fig. 2. A slight tendency towards larger displacement values with longer PVC coupling interval was observed (p = 0.252). Patient age, BMI, sex, antiarrhythmic medication, left-ventricular ejection fraction and the mapped cardiac chamber had no influence on spatial displacement (Table 2). The spatial displacement had no effect on total ablation energy or procedure time.

**Table 2:** Predictive variables and their influence on spatial displacement

Variable	Regression coefficient (mm)	95% CI	p-value
Age (per 10 years increase)	− 0.06	(− 0.63, 0.53)	0.855
BMI (per point increase)	0.05	(− 0.11, 0.20)	0.559
Sex	0.10	(− 1.40, 1.60)	0.892
Impairment of left-ventricular ejection fraction			
Mild	− 0.43	(− 2.10, 1.22)	0.598
Moderate	− 0.86	(− 2.78, 1.06)	0.365
Severe	− 0.74	(− 2.87, 1.39)	0.479
Antiarrhythmic medication	0.56	(− 1.39, 2.50)	0.563
Coupling interval (per 100 ms increase)	0.48	(− 0.36, 1.32)	0.252
Area of PVC-origin (outflow-tract vs. non-outflow-tract)	− 1.33	(− 2,63, − 0.03)	0.045*
Mapped chamber	0.56	(− 0.93, 2.05)	0.445

Predictive variables and their respective influence on median spatial displacement. For continuous variables, the linear regression coefficient and its 95% confidence interval is displayed. We observed a significant influence for the area of PVC-origin

*p ≤ 0.05

To evaluate the influence of the mapping point’s exact location on its spatial displacement, a subanalysis on three anatomically complete maps was performed. We chose PVCs with a similar area of earliest activation to minimize the effect of PVC-origin on spatial displacement. The maps were comprised of 1310 hybrid mapping points. Two maps were obtained from the right ventricle and one map from the left ventricle. All patients suffered from structural heart disease (dilated cardiomyopathy/ischemic cardiomyopathy) and showed PVCs originating from the septal myocardium. The results are shown in Table 3.

**Table 3:** Subanalysis of 3 anatomically complete hybrid activation maps

Location	Number of points	Median displacement (mm)	IQR (mm)	p-value
Total	1310	2.9	1.9–4.1	–
Right ventricle	938	3.0	1.9–4.2	0.13
Free wall	98	3.3	2.0–4.3	0.04 *
Inferior wall	242	2.9	2.1–3.6	0.47
Septum	492	2.9	2.0–4.5,	0.15
RVOT	106	3.0	1.4–4.3	0.32
Left ventricle	372	2.8	1.9–3.9	0.13
Free wall	49	3.9	2.9–5.2	< 0.0001***
Inferior wall	89	2.4	1.7–3.6	0.006**
Septum	207	2.7	1.8–3.8	0.007**
LVOT	27	3.6	2.1–5.8	0.07
Outflow-tract	133	3.3	1.6–4.4	0.96
Ventricle	1139	2.9	1.9–4.0	0.14

IQR: interquartile range, RVOT/LVOT: right/left ventricular outflow tract. Two-sided p-values were calculated via Mann–Whitney-U-test

Findings that reached statistical significance are marked with asterisks (*p ≤ 0.05; **p ≤ 0.01; ***p ≤ 0.001)

In total, we observed a comparatively low median spatial displacement of 2.9 (IQR 1.9–4.1) mm for all mapping points. There were no significant differences in spatial displacement between outflow-tracts and ventricle in general (p = 0.96).

Indeed, we observed significant differences between distinct locations within the ventricular myocardium. The displacement was significantly wider for points acquired in the RV free wall and the LV free wall (Table 3). Conversely, significantly lower values for spatial displacement were recorded for the inferior and septal ventricular walls. While a trend towards more displacement in the LVOT was observed, the small number of points (n = 27) does not allow a conclusive statement.

## Discussion

For catheter ablation of PVCs, accurate mapping is essential for correct localization of ablation targets and efficient suppression of PVCs. Even small inaccuracies in mapping might lead to unsuccessful ablation attempts, which may induce edema in the myocardial area of interest and complicate further ablation attempts [10]. The main finding of this study was that the spatial displacement of mapping points, determined by movement of the catheter tip during PVC compared to the preceding sinus beat, is larger in activation mapping of non-outflow tract PVCs compared to outflow tract PVCs. This has implications for mapping procedures targeting e.g., the Purkinje system and the LV wall including papillary muscles. Hybrid activation mapping is a novel tool to correct for spatial displacement of the mapping catheter during premature ventricular contractions (PVC). First clinical results have been published [8], but more data is needed to thoroughly assess the myocardial displacement and the factors influencing it.

Our results show that the average mapping point is displaced by ca. 4 mm, with a 33% larger displacement for non-outflow tract PVCs. The extent of spatial displacement observed in our study is supported by the observation of  Steyers et al. in their study about 21 patients and 606 mapping points. The authors found a mean displacement of 4.4 ± 2.4 mm [9]. Andreu et al. described a median spatial displacement of 9.42 mm (IQR 6.19–12.85) in a study including 55 patients, analyzing 6923 mapping points [7]. In this study, the authors manually corrected for the shift by re-annotating each point to the location of its preceding sinus rhythm beat. De Potter et al. found a mean spatial displacement of 8.9 ± 5.5 mm in a small sample of 127 hybrid points using automatic correction for spatial displacement [8].

The cranial portions of the heart containing the outflow tracts and the valvular plane are more fixed by adjoining structures like the great vessels in comparison to non-outflow-tract myocardium, which is surrounded by the pericardial space [7]. Their physiological displacement is less prominent than in the apical portions or the free wall of the ventricles. This might be a factor limiting the extent of spatial displacement in PVCs originating from the ventricular outflow tracts, especially in patients with preserved ventricular function. Additionally, parts of the outflow tracts are located close to the AV-node and His-bundle. Ectopic electrical activity might therefore enter His-purkinje-system earlier than in non-outflow tract PVCs. The resulting myocardial contraction might resemble the physiological contraction more closely, contributing to a smaller spatial displacement. Similarly, small differences of spatial displacement between mapping points located in different myocardial areas were observed in an earlier study [7]. However, the authors differentiated between location of mapping points, while we differentiated between different areas of PVC-origin. The aforementioned anatomical conditions for the outflow tracts might play a role for both findings. However, an earlier entry of electrical activity into the His-purkinje-system is influenced by PVC-origin, not on the sole location of mapping points. Therefore, the results are not entirely comparable.

Reported data concerning influencing factors for spatial displacement are heterogeneous:

Since movement of the myocardium during diastolic filling is expected, the phase of the cardiac cycle in PVC and in sinus rhythm on which the point is acquired, and which is dependent on the coupling interval, represents an important factor in our analysis.

Steyers et al. reported a trend towards larger displacement with a lower PVC-coupling interval that did not reach statistical significance, while this correlation reached statistical significance in the work published by Andreu et al. [7, 9]. In contrast, we saw a slight trend towards larger displacement for PVCs with a longer coupling interval. The difference in the reported results could be explained by disparities in the patient collectives: The vast majority of patients in the study of Andreu et al. had PVCs originating from the ventricular outflow tracts [7], while the origin of PVCs was distributed almost equally between ventricular outflow tracts and non-outflow tract myocardium in our study population. Since the ventricular outflow tracts are more fixed by adjoining vessels, inadequate ventricular filling associated with a shorter coupling interval might be more relevant for spatial displacement for PVCs originating in the cranial portions of the heart [7]. With a short coupling interval, the ectopic activity commences early in the diastole with a short time for relaxation and ventricular filling after the preceding sinus beat, leading to a lower stroke volume and inotropy for the premature beat. It is therefore conceivable that in our population with more non-outflow-tract PVCs, the better ventricular filling and inotropy associated with a longer coupling interval might accentuate the spatial displacement as one can expect a more unphysiological contraction sequence in non-outflow-tract origins.

The previously reported influence of the mapped heart chamber on spatial displacement [7] could neither be confirmed by our study nor by Steyers et al. [9]. Again, this discrepancy might be explained by differences in the study populations: PVCs originating outside the outflow tracts are associated with structural heart disease [11], which was more prevalent in the left ventricle in our study population. That these PVCs showed significantly more displacement, might balance out the association between spatial displacement and mapped chamber previously reported for PVCs originating around the valvular plane [7].

The subanalysis yields interesting results that nicely demonstrate the multifactorial genesis of the spatial displacement: The free walls of both ventricles are prone to a wider displacement, which may be explained by the fact that they are not fixed to adjoining structures and therefore have a low resistance to movement. The inferior wall and the ventricular outflow tracts are more constrained by large vessels and fixation on the diaphragm, leading to a lower spatial displacement during a PVC.

However, the comparatively low displacement values for these maps in general cannot solely be explained by cardiac anatomy. The fact that all maps showed a septal PVC-origin is in line with our considerations that the point of origin also has an influence on spatial displacement: More constrained locations of origin close to the His-purkinje-system show lower displacement in general.

Although first clinical results show the efficacy of catheter ablation using hybrid mapping [8, 9, 12], a clinical benefit compared to conventional activation mapping is yet to be demonstrated. Since standard ablation catheters have a 3- to 4-mm tip, it seems plausible that a correction for spatial displacement might improve the accuracy of RFC-delivery in a clinically significant extent. According to our results this might be especially relevant for non-outflow tract PVCs, as those showed a significantly larger displacement. With broader use of the novel mapping software, we can expect to learn more about the spatial displacement of mapping points in PVCs. A randomized, controlled study comparing results of ablation procedures with hybrid activation mapping against procedures with conventional activation mapping would be desirable to evaluate the potential clinical benefit of the novel mapping feature.

## Limitations

In some maps, singular points with a very high value for spatial displacement were observed. Since even small catheter displacement is supposed to be recorded, stability filters are disabled during hybrid mapping. Therefore, movement of the catheter between PVC and the preceding sinus beat may lead to false-high values for spatial displacement. Although we carefully checked all annotations during the procedure, singular false-high measurements for spatial displacement cannot be completely ruled out. Since we used median values for our analyses, we believe this bias to be of minor relevance for our results.

Secondly, even though our analysis covered a total of 5798 individual mapping points, the studied patient population of 22 constitutes a small sample size. Thirdly, all analyzed data was recorded using the CARTO system. Although the described spatial displacement should be detectable irrespective of the mapping system, small differences between the platforms cannot be ruled out.

There are several possible factors influencing the spatial displacement of myocardium during PVCs. Whether changes in diastole or systole are the dominant factors cannot be answered conclusively by the here presented study.

Lastly, without a control group, the effect of spatial displacement on clinical outcome cannot be evaluated with certainty based on this study.

## Conclusion

Ectopic activation of the ventricular myocardium during PVCs results in a spatial displacement of mapping points. We observed a mean spatial displacement of 3.8 ± 1.5 mm that was dependent on the location of PVC-origin: mapping points of PVCs with non-outflow-tract origin showed a larger displacement than PVCs originating from the outflow tracts. The correction for spatial displacement may help to improve accuracy of RFC-delivery in catheter ablation of PVCs. Our results suggest that this is especially relevant for PVCs with non-outflow-tract origin.

## Data Availability

Anonymized mapping data and demographic parameters were provided to the editor. Raw mapping data is stored on an internal server due to data protections guidelines. Raw anonymized mapping data is available from the corresponding author upon reasonable request. A preprint version of this work is available online (https://doi.org/10.21203/rs.3.rs-594511/v1).
